# Antenatal depression and its potential causal mechanisms among pregnant mothers in Gondar town: application of structural equation model

**DOI:** 10.1186/s12884-020-02859-2

**Published:** 2020-03-17

**Authors:** Abel Fekadu Dadi, Emma R. Miller, Richard Woodman, Telake Azale Bisetegn, Lillian Mwanri

**Affiliations:** 1 Department of Epidemiology and Biostatistics, Institute of Public Health, College of Medicine and Health Sciences, University of Gondar, Gondar, Ethiopia; 2 College of Medicine and Public health, Discipline of Public health, Flinders University, Health Sciences Building, Sturt Road, Bedford Park, Adelaide, SA 5001 Australia; 3 College of Medicine and Public health, Center for Epidemiology and Biostatistics, Health Sciences Building, Sturt Road, Bedford Park, Adelaide, SA 5001 Australia; 4 Department of Health promotion and Behavioral sciences, Institute of Public Health, College of Medicine and Health Sciences, University of Gondar, Gondar, Ethiopia

**Keywords:** Antenatal depression, Pregnant mothers, Structural equation modeling, Stressor

## Abstract

**Background:**

Various forms of life stressors have been implicated as causes of antenatal depression. However, there is a lack of understanding of which forms of stress lead to antenatal depression and through what mechanisms. Modeling stress processes within a theoretical model framework can enhance an understanding of the mechanisms underlying relationships between stressors and stress outcomes. This study used the stress process model framework to explore the causal mechanisms underlying antenatal depression in Gondar, Ethiopia.

**Methods:**

Questionnaires, using an Online Data collection Kit (ODK) tool were administered face-to-face in 916 pregnant women in their second and third trimesters. Pregnant women were included from six randomly selected urban districts in Gondar, Ethiopia during June and August 2018. The Edinburgh Postnatal Depression Scale (EPDS) was used to screen for antenatal depression. A Structural Equation Model (SEM) was employed to explore the direct, indirect, and total effect of stressors and mediators of antenatal depression.

**Result:**

Sixty-three participants (6.9%) reported symptoms of depression. Of these, 16 (4.7%) and 47 (8.1%) were in their second and third trimesters, respectively. The SEM demonstrated several direct effects on antenatal depression scores including unplanned pregnancy (standardized β = 0.15), having a history of common mental health disorder (standardized β = 0.18) and fear of giving birth to the current pregnancy (standardized β = 0.29), all of which were associated with a higher depression score. Adequate food access for the last 3 months (standardized β = − 0.11) was associated with decreased depression score. Social support (β = − 0.21), marital agreement (β = − 0.28), and partner support (β = −.18) appeared to partially mediate the link between the identified stressors and the risk of antenatal depression.

**Conclusion:**

Both direct and indirect effects contributed to higher antenatal depression score in Ethiopian women. The three psychosocial resources namely marital agreement, social and partner support, mediated reduced antenatal depression scores. Early screening of antenatal depression and enhancing the three psychosocial resources would help to improve maternal resilience.

## Background

Pregnancy is an important period of vulnerability for depression, which has been associated with hormonal and/or biological changes [1, 2] and receives differential health care support across populations [3]. Antenatal depression can be accompanied by signs and symptoms of low mood, tiredness, insomnia, lack of energy, forgetfulness, irritability, and poor physical and cognitive functioning [4]. Antenatal depression manifests at different times [5] including an increase in the early and late weeks of pregnancy and a decrease in the middle of the pregnancy [6].

Systematic reviews of studies conducted in developed countries reported prevalence in the range of 5–30%, with variation by socio-demographic, obstetric, and measurement related factors [7–9]. Using the Edinburgh Postnatal Depression Scale (EPDS) as a screening tool, antenatal depression prevalence ranged from 10.4 to 57% in lower income Asian countries [10–20] and between 22.7 and 38.5% amongst African countries [21–24]. In Ethiopia, prevalence ranges from 11.8 to 31.12% with variation according to the type of screening tool and study setting [25].

Depression during pregnancy can negatively affect fetal growth and lead to poor outcomes including preterm birth, low birth weight [26, 27], poor fetal brain development [28], poor coping ability in the child’s later life [29] and, especially in combination with partner violence to an increased risk of child death [30]. Similarly, antenatal depression has been associated with reduced maternal health care service uptake, a reduced ability to care for the newborn [31], increased substance use, poor appetite [32–34], suicide [35, 36] and a negative impact on fetal immune development [37]. Intergenerational effects have also been reported indicating that children born from depressed mothers were more likely to have depression during their own adolescence and motherhood [38].

Modeling of the stress process within a theoretical model framework can assist in understanding the various pathways and mechanisms underlying the relationships between stressors and stress outcomes [38, 39]. Although a number of antenatal depression studies have been conducted in Ethiopia [40–43], they did not examine the mechanims underlying the relationship between potential causal factors and antental depression. Hence, additional studies are needed to determine the possible mechanisms underlying antental depression. Using a stress process model framework might give further insight towards prevention and control of depression during pregnancy [44].

**Fig. 1:**
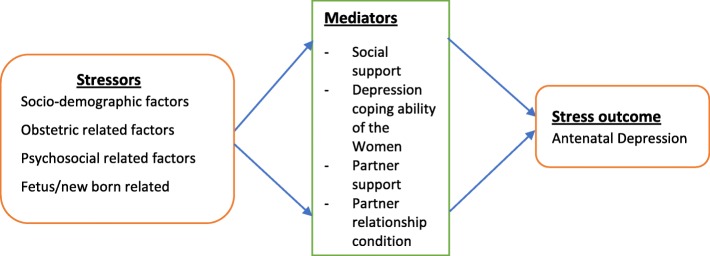
A stress process model, a theoretical frame work, adapted from Pearlin et al. 1981

The stress process model was developed by Peirlin and colleagues [45] and consists of three main conceptual domains: the source of stress (stressor) domain, which includes life events and chronic life strains; the mediators domain, which includes any mediators of stress that have an ability to mediate the impact of stressfull situations such as social support and coping styles; and the manifestation of the stress or stress outcomes as various mental disorders. This stress process model has been tested both in pregnant [46] and postnatal [47] populations in China for antenatal and postnatal specific depression. For the current study we considered direct stressors including sociodemographic factors, obstetric and psychosocial factors and mediating factors such as social and personal resources (social support, partner support, personal coping abilities). (Fig. 1). As such, this study used the stress process model framework to explore the causal mechanisms underlying antenatal depression in Gondar, Ethiopia.

## Methods

### Study setting

The current study was conducted in Gondar town, which is one of the administrative zones of Amhara Regional State, Northwest Ethiopia. Gondar town is in the Northern part of the Amhara region at 747 km away from Addis Ababa and 170 km from Bahirdar (the regional capital city). Gondar town has 12 kebeles (the smallest administrative units in the country) and in 2017/2018, the number of pregnancies in the town was expected to be 6450 [48, 49]. The town has one government-operated referral hospital, more than eight health centers, and more than 15 private medical clinics [50].

### Study population

The study population included pregnant women living in the randomly selected districts and in their second and third trimester of pregnancy. A house-to-house visit was conducted to identify pregnant women who were willing to participate in this mother- child cohort study. If nobody was found at home during the initial recruitment visit and after three attempts, they were non-respondents. Identified participants were recruited and were followed until 12 weeks post-delivery.

### Ethical approval

Ethical approval was obtained from the Social and Behavioral Research Ethics Committee (SBREC) of the Flinders University [51] and the Institutional Review Board of University of Gondar. A support letter was obtained from Gondar town mayoral office and the respective kebeles administration offices. Participants of the study were informed about the purpose, objectives, their right to decline participation or withdraw their participation. A written consent was then obtained. Privacy and confidentiality were maintained throughout the study. Women who were found to be seriously ill and fulfilled the following criteria were referred to University of Gondar Specialized Hospital Psychiatry unit for further diagnosis and treatment: an overall Edinburgh Postnatal Depression Scale (EPDS) score of 13 (those with ≥17 were excluded from the study for further follow up) and those who had a score 1, 2, 3 on item ten (a question about thoughts of self-harm) [52].

### Data collection and the questionnaire

Structured and pre-tested electronic questionnaires were administered face-to-face in pregnant women aided by an online, Open Data collection Kit (ODK) application tool [53]. Open data collection kit is an application developed by the ODK community for collecting, managing, and using data in resource limited countries [54]. The prepared questionnaire was designed on an excel spreadsheet, converted to XLS format online, and checked for its validity using Enketo (a preview provided by ODK). The validated form was uploaded on a Lenovo 7 tablet. During collection, data were stored on the Google cloud platform. Nine qualified and registered nurses were trained as data collectors and were each provided with a Lenovo 7 tablet to administer the questionnaire to the participants. After completion of each questionnaire, the data collectors uploaded the data to Google Cloud and the principal investigator then directly downloaded the data from the system.

The electronic based data collection was helpful in maintaining the quality and completeness of the data. The questionnaire collected socio-demographic information such as: age; sex; educational status (no formal education, grade 1–8, grade 9–12, diploma and above); income (low, medium, high); and marital status (single, married, separated). Information on maternal characteristics was also collected, including pregnancy intention (planned, unplanned); gestational weeks; previous history of either low weight, preterm or still birth; and previous history of a caesarian section delivery. Finally, the questionnaire collected information on psychosocial and behavioral characteristics, such as: social support (good, poor); partner support (always, most of the time, some of the time, rarely); stress coping ability (very rarely, rarely, sometimes, most of the time); coffee drinking (daily, sometimes, never); and cigarette exposure (yes, no).

### Instruments

Antenatal depression was measured using the Edinburgh Postnatal Depression Scale (EPDS) developed by Cox and colleagues [52] and adapted for use in an Ethiopian context [55]. The EPDS, which is the most commonly used screening tool for antenatal depression [56–60], is a brief screening tool for symptoms of emotional distress during pregnancy that contains 10 specific questions with four Likert scale response options (most of the time, sometimes, not often, never) and is intended to measure the distress that pregnant women have experienced over the previous week. It is a simple and free to use tool, can be scored by simple addition and has been validated in urban settings of Ethiopia [61] with a sensitivity and specificity of 84.7 and 77.0%, respectively. The validated cut off value for possible depression in urban population in Ethiopia was 12 [43, 62, 63]. In the current study the EPDS demonstrated high reliability for the single construct of distress with an internal consistency (α) of 0.74.

The Oslo Social Support Scale (OSSS-3) [64] was used to measure maternal social support during pregnancy. Although the tool has not been validated in the Ethiopian context, it has shown a good utility in various studies [62, 65]. OSSS-3 has three items measured by a few Likert scales, which are summed to 14 points and categorized as ‘poor’ if the total score is less than nine and ‘moderate’ to ‘strong’ support if the score is 9–14. In this study OSSS-3 demonstrated a high reliability for social support with an internal consistency of α = 0.76. Partner support was assessed by a question “My husband helps me a lot” with five response scales, “Always”, “Most of the time”, “Some of the time”, “Rarely”, and “Never”. Marital agreement was assessed by a question “How often do you discuss and agree with your husband in day to day life?” with a response category, “Most of the time”, “Some of the time”, “Rarely”, and “Never”.

The women’s Middle-Upper Arm Circumference (MUAC) tape was used to measure nutritional status. MUAC is a validated and recommended tool for measuring nutritional status during pregnancy, with cutoff scores of 18–22 as ‘normal’ and 22.5 to 31 as ‘underweight’ [66]. Women were asked if they participated in moderate-intensity physical activity such as brisk walking, dancing, gardening, and usual housework for 2 to 3 h per week [67]. Exposure to cigarette smoking during pregnancy was assessed by the question, “Have you been smoking since your pregnancy or has there been anybody who smokes near you in your home or in your workplace?” [15]. To assess coffee exposure, we asked “How often do you drink coffee after your pregnancy?” if her answer was “daily” or “sometimes in a week”, she was labeled as exposed to coffee drinking and if not, she was labeled as non-exposed [68, 69]. Women’s health condition was assessed using the question “How do you rate your daily general health condition?” with response options of “Very good”, “Good” or “Poor”.

A women’s stress coping level was assessed using the four customized internally-consistent coping subscales of the Perinatal Coping Inventory (PCI-4), which was specifically developed for pregnancy [70]. Coping styles within this tool included: (1) Preparation for motherhood, “planned how you will handle the birth” (2) Avoidance “avoided being with people in general” (3) Positive appraisal “felt that being pregnant has enriched your life” and (4) Prayer “prayed that the birth will go well”. Women were asked to report how often they used each of the above coping styles and their response was recorded using a 4-point Likert scale; 0 (Never), (1) rarely, (2) sometimes, (3) most of the time [71]. In this study, PCI-4 demonstrated a moderate reliability with an internal consistency of α = 0.50.

### Sample size

The sample size calculation was based on the estimated effect of perinatal depression on adverse infant health outcomes. To calculate this, we used a double population proportion formula in Epi Info version 7 [72] with the following assumptions: 95% confidence level, 90% power, an exposed to non-exposed ratio of 1:2, a prevalence of underweight among those free from common mental disorder of 25%, and a difference of 1.5. A total sample size of *n* = 809 was estimated which was then increased by 20% to account for expected losses to follow up. The final sample size was therefore estimated as *n* = 970.

### Statistical analysis

Completed data were downloaded from the Google Cloud platform in Excel spreadsheet form, checked for completeness and imported to Stata version 14 (StataCorp, USA) for further cleaning and analysis. Descriptive statistics included mean, median, proportion/percentage, interquartile range, and standard deviations as appropriate. A chi-squared test was used to test for crude associations between the categorical stressors and evidence of depression based on a cut of score of 12. A Structural Equation Model (SEM) was constructed that reflected the stress-process model framework and which explored the direct and indirect relationships between the independent (stressors) and the dependent (antenatal depression) variables. This allowed us to assess the strength of the hypothesized direct and indirect causal paths [73, 74].

In order to better fit the measurement model for depression, the measurement items for the depression scale were parceled into three categories using a random based parceling algorithm. Parceling allows for recategorizing multiple items of a scale in order to get better model fit and convergence [75, 76]. The first parcel contained the EPDS items 1, 4, and 9. The second parcel contained the EPDS items 6, 7, and 8. The third parcel contained the EPDS items 2, 3, 5, and 10. Since the subsequent parcels displayed evidence of non-normality we used the Satorra-Bentler scaled chi-squared test when estimating model fit since this is robust to non-normality [77].

**Fig. 2:**
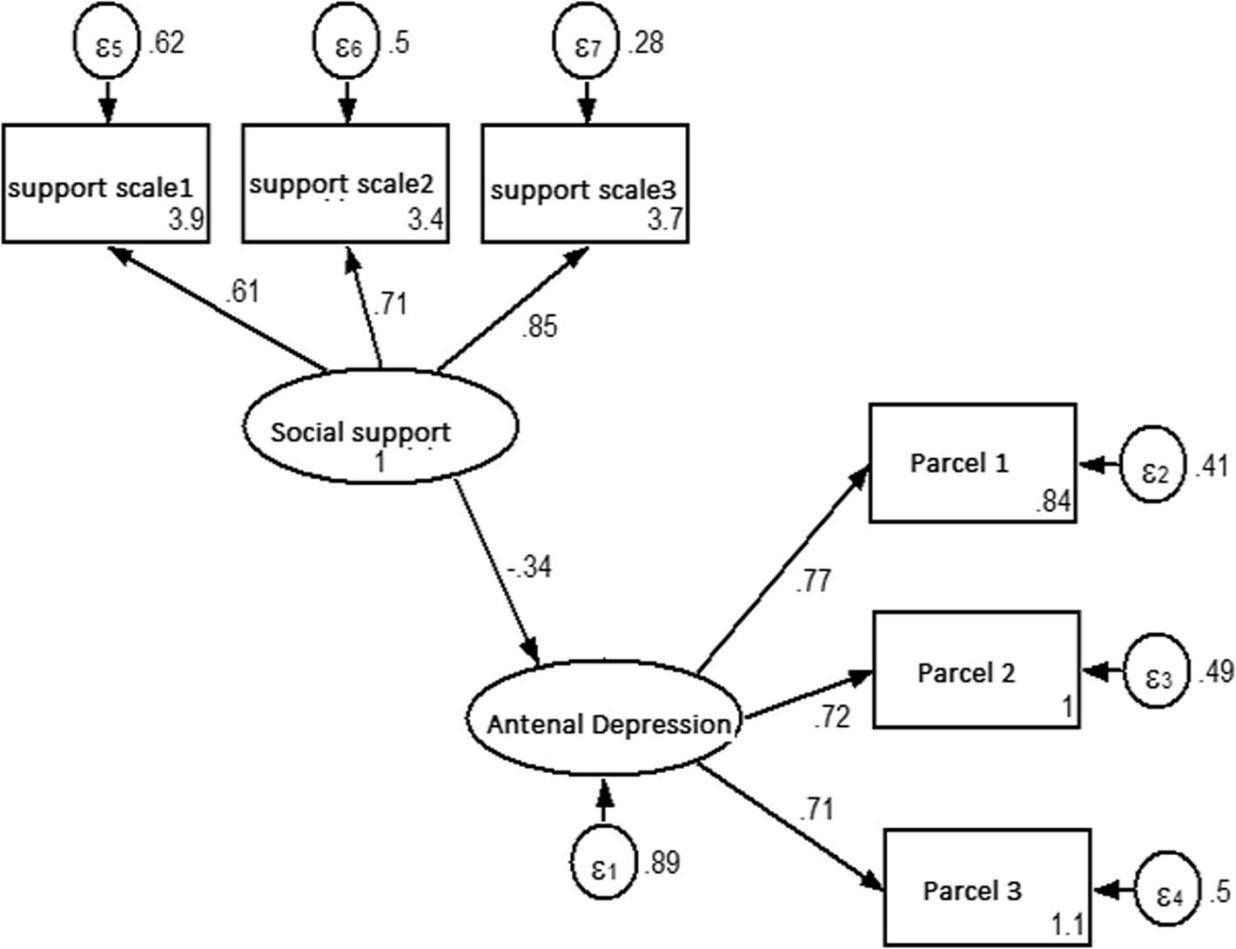
Measurement model for elucidating the stress process for antenatal depression (*N* = 916), Gondar town, Ethiopia, 2018

The potential stressors and hypothesized causal paths were selected based on prior subject knowledge (which informed the questionnaire). In addition, a multivariate mixed effects regression analysis was performed to help determine variables suitable for inclusion in the SEM conditioning for socio-demographic, maternal obstetrics and psychosocial factors that were significantly associated (*P* < 0.05) in bivariate mixed effects regression. Stressors included income, attitude to the current pregnancy, history of common mental disorders, and a fear of giving birth. Potential mediators for the association included social support, marital agreement and stress coping. Figure 2 shows the hypothesized causal pathways. Model fit was assessed for the hypothesized model and an iterative approach was used to modify the model through adding and removing paths until a theoretically supported and a statistically well-fitted model was obtained. A model that was over identified, recursive, simple, theoretically meaningful, and the best fit for the data was retained and interpreted [78].

The overall fit of the model was assessed using the following tests and recommended limits: Sartorra-Bentler Chi-squared test of fit (*p* > 0.05); Tucker Lewis Index (TLI) and Comparative Fit Index (CFI) value ≥0.90; and Root Mean Square Error of Approximation (RMSEA) ≤ 0.08 [79]. The direct, indirect, and total effects of the stressors on antenatal depression were reported in the form of standardized beta coefficients. Estimated effects for which *p* < 0.05 were considered as being statistically significant.

## Results

Of 960 pregnant women contacted in the six selected kebeles, 15 refused to provide consent for participation in the study and five were unavailable after three further attempts to contact them, leaving 940 women who agreed to participate, at a 95.4% response rate. After the initial screening, 24 participants with an EPDS of ≥17 were excluded due to a high likelihood of having serious depression, and the potential to providing a non-informed consent. Following these exclusions, a total of 916 participants were included in the analysis. Sixty-three participants had an EPDS score between 12 and 16, giving a prevalence of possible depression of 6.9%.

### Socio-demographic characteristics of the participants

Table 1 describes the characteristics of the study population according to their risk of depression as assessed by the EPDS scale. The mean (±SD) age of the participants was 26.5 years (±4.5) and the majority (61.2%) were in aged between 25 and 34-years. There was no mean age difference between participants with and without signs of depression (EPDS score ≥ 13). The mean (±SD) monthly income of the participants was 3496.5 (±2962.3) Ethiopian Birr. The mean income of participants with depression was lower than those without possible depression (*p* < 0.01). There were 347 (37.8%) participants who attained secondary education and 654 (71.4%) were undertook unpaid home duties. There were 441 (49.2%) participants who discussed and agreed on things with their partners most of the times with a significant association between the marital situation and depression status (*p* < 0.001). Most of the participant (95.8%) had poor access to food in their household in the previous 3 months and there was an association between poor access to food in the last 3 months and depressive symptoms (*p* < 0.001).

**Table 1:** Socio-demographic characteristics of participants included in the study (*N* = 916), Gondar town, Ethiopia, 2018

Variable/category	Risk of depression			
	Had depression (*n* = 63), n (%)	No depression (*n* = 853), n (%)	Total *n* = 916, n (%)	*p*-value
Mothers’ age				0.707
18–24	20 (31.8)	271 (31.8)	291 (31.8)	
25–34	37 (58.7)	524 (61.4)	561 (61.2)	
> =35	6 (9.5)	58 (6.8)	64 (7.0)	
Mean(±SD)	26.9 (±5.2)	26.5(±4.5)	26.5(±4.5)	
Household monthly income				0.057
Low	40 (63.5)	409 (48.0)	449 (49.0)	
Medium	19 (30.2)	355 (41.6)	374 (40.8)	
High	4 (6.3)	89 (10.4)	93 (10.2)	
Monthly income (Mean(±SD)	2753 (±2160)	3551.4(±3006)	3496.5(±2962.3)	
Mothers’ education				0.024
No formal education	12 (19.1)	106 (12.4)	118 (12.9)	
Grade 1–8	13 (20.6)	221 (25.9)	234 (25.6)	
Grade 9–12	31 (49.2)	316 (37.1)	347 (37.8)	
Diploma and above	7 (11.1)	210 (24.6)	217 (23.7)	
Mothers’ occupation				0.016
Home duties	42 (66.7)	612 (71.7)	654 (71.4)	
Student	2 (3.2)	13 (1.5)	15 (1.6)	
Government employee	4 (6.3)	125 (14.7)	129(14.1)	
Self-employee	15 (23.8)	103 (12.1)	118 (12.9)	
Mothers’ religion				0.848
Orthodox	51 (80.9)	683 (80.1)	734 (80.1)	
Muslim	11 (17.5)	162 (19.0)	173 (18.9)	
Protestant	1 (1.6)	8 (0.9)	9 (1.0)	
Mothers’ marital status				0.001
Single	5 (7.9)	11 (1.3)	16 (1.8)	
Married	54 (85.7)	826 (96.8)	880 (96.1)	
Divorced	1 (1.6)	3 (0.4)	4 (0.4)	
Separated	3 (4.8)	13 (1.5)	16 (1.7)	
How often partner discuss and agree on things from their own perspectives				0.001
Most of the time	13 (22.8)	428 (51.0)	441 (49.2)	
Sometimes	25 (43.9)	352 (42.0)	377 (42.1)	
Rarely	15 (26.3)	52 (6.2)	67 (7.5)	
Never	4 (7.0)	7 (0.8)	11 (1.2)	
Difficulty to access food for their family in the last 3 months				0.001
Yes	13 (20.6)	25 (2.9)	38 (4.2)	
No	50 (79.4)	828 (97.1)	878 (95.8)	

*p*-value is based on Chi-square or fisher exact test wherever the expected cell counts are < 5

### Maternal characteristics of the participants

Table 2 shows the maternal and obstetric characteristics of the study participants. Most pregnancies (84.8%) were planned. A lower proportion of planned pregnancies were in participants with signs of depression (49.2%) than those without (87.5%) (*p* < 0.001). Sixty-three percent of pregnancies were in their third trimester and the mean (±SD) pregnancy gestation period was 27.8 (±6.7) weeks. Three hundred and forty-nine (38.1%) women were pregnant for the first time and the mean (±SD) parity was 2.1 (±1.2). Of participants who had two or more children, 93.5, 96.5 and 87.4% had no history of preterm, low birthweight, or cesarean section delivery respectively. Participants with signs of depression were more likely to have previously experienced preterm birth (*p* = 0.001). Seven hundred and twenty-one women (78.7%) feared giving birth in their current pregnancy. The mean (±SD) mid-upper arm circumference was 24.1(±1.7) cm, with 130 (14.2%) underweight. Participants with signs of depression were more likely to have a fear of giving birth (p = 0.001), were exposed to cigarette smoking (*p* = 0.003) and were less likely to undertake physical activity (*p* = 0.009).

**Table 2:** Maternal and obstetric characteristics of the study participants (*N* = 916), Gondar town, Ethiopia, 2018

Variable/category	Risk of depression		Total *n* = 916, n (%)	p-value
	Had depression (*n* = 63), n (%)	No depression (*n* = 853), n (%)		
Pregnancy condition				0.010
Planned	31 (49.2)	746 (87.5)	777 (84.8)	
Un-timed	26 (41.3)	97 (11.4)	123 (13.4)	
Un-intended	6 (9.5)	10 (1.1)	16 (1.8)	
Pregnancy trimester				0.170
2nd trimester	16 (37.9)	323 (25.4)	339 (37.0)	
3rd trimester	47 (62.1)	530 (74.6)	577 (63.0)	
Pregnancy weeks (mean(±SD))	28.8(±5.8)	27.7(±6.7)		
Difficulty to conceive in the current pregnancy				0.067
Yes	9 (14.3)	66 (7.7)	75 (8.2)	
No	54 (85.7)	787 (92.3)	841 (91.8)	
Parity of the mother				0.450
1	24 (38.1)	325 (38.1)	349 (38.1)	
2	16 (25.4)	272 (31.9)	288 (31.4)	
3–8	23 (36.5)	256 (30.0)	279 (30.5)	
Mean(±SD)	2.3(±1.4)	2.1(1.2)	2.1(±1.2)	
Preterm history				0.001
Yes	8 (20.5)	29 (5.5)	37 (6.5)	
No	31 (79.5)	499 (94.5)	530 (93.5)	
Low birth history				0.144
Yes	3 (7.7)	17 (3.2)	20 (3.5)	
No	36 (92.3)	511 (96.8)	547 (96.5)	
History of cesarean section				0.981
Yes	5 (12.8)	67 (12.7)	72 (12.7)	
No	34 (87.2)	461 (87.3)	495 (87.3)	
Antenatal care service uptake (at least one)				0.069
Yes	57 (90.5)	815 (95.5)	872 (95.2)	
No	6 (9.5)	38 (4.5)	44 (4.8)	
Had fear of birth				0.001
Yes	37 (58.7)	158 (18.5)	195 (21.3)	
No	26 (41.3)	695 (81.5)	721 (78.7)	
Partner had different sex preference				0.066
Yes	23 (36.5)	221 (25.9)	244 (26.6)	
No	40 (63.5)	632 (74.1)	672 (73.4)	
Did physical activity				0.009
Yes	59 (93.7)	839 (98.4)	898 (98.0)	
No	4 (6.3)	14 (1.6)	18 (2.0)	
Mothers health condition in this pregnancy from their own perspective				0.001
Very good	10 (15.9)	269 (31.5)	279 (30.5)	
Good	43 (68.2)	536 (62.9)	579 (63.2)	
Bad	10 (15.9)	48 (5.6)	58 (6.3)	
Exposure to cigarette				0.003
Yes	14 (22.2)	86 (10.1)	100 (10.9)	
No	49 (77.8)	767 (89.9)	816 (89.1)	
Exposure to coffee				0.078
Daily	29 (46.0)	351 (41.1)	380 (41.5)	
Sometimes	26 (41.3)	287 (33.7)	313 (34.2)	
Never	8 (12.7)	215 (25.2)	223 (24.3)	
Nutritional status of the mother				0.982
Normal (MUAC 18–22)	54 (85.8)	732 (85.7)	786 (85.8)	
Underweight (MUAC 22.5–31)	9 (14.2)	121 (14.3)	130 (14.2)	
MUAC (Mean(±SD)	23.9(±1.5)	24.1(±1.7)		

*p*-value is based on Chi-square or fisher exact test wherever the expected cell counts are < 5

### Psycho-social characteristics of the participants

Table 3 displays the psychosocial characteristics of the participants. The majority (93.1%) of participants had no history of common mental health disorders. However, compared to non-depressed participants, a higher percentage of women in the depressed group reported a history of common mental health disorders (*p* = 0.001). Seven-hundred and eighty-five (80.2%) participants reported good social support and 420 (46.9%) reported receiving support from their partners. Participants in the depressed group had a lower social or partner support compared to the non-depressed group (p = 0.001). Five-hundred fifty-eight (60.1%) participants reported they rarely coped with their stress.

**Table 3:** Psycho-social characteristics of the study participants (*N* = 916), Gondar town, Ethiopia, 2018

Variable/category	Risk of depression		Total *n* = 916, n (%)	P-value
	Had depression (*n* = 63), n (%)	No depression (*n* = 853), n (%)		
History of common mental disorder				0.001
Yes	11 (17.5)	52 (6.1)	63 (6.9)	
No	52 (82.5)	801 (93.9)	853 (93.1)	
Social support				0.001
Good	36 (57.1)	699 (81.9)	785 (80.2)	
Poor	27 (42.9)	154 (18.1)	181 (19.8)	
Social support scale (Median(±IQR))	9(8–12)	11(9–13)		
Internal consistency (α)	0.76 (high reliability)			
Partner support				0.001
Always	17 (29.8)	403 (48.0)	420 (46.9)	
Most of the time	11 (19.3)	254 (30.3)	265 (29.6)	
Some of the time	20 (35.1)	150 (17.9)	170 (19.0)	
Rarely	9 (15.8)	32 (3.8)	41 (4.5)	
Coped with stress				0.042
Very rarely	2 (3.2)	5 (0.6)	7 (0.8)	
Rarely	31 (49.2)	527 (61.8)	558 (60.9)	
Sometimes	26 (41.3)	277 (32.5)	303 (33.1)	
Most of the time	4 (6.3)	44 (5.1)	48 (5.2)	
Stress coping scale (Median (IQR)	8(7–10)	8(7–10)		
Internal consistency (α)	0.5 (moderate reliability)			
Symptom of Depression (EPDS 12–16)				
Yes	63 (6.9%; 95%CI: 5.3, 8.7%)			
No	853 (93.1%)			
Depression scale (Median(±IQR))	4(0–5)			
Internal consistency (α)	0.74 (High reliability)			

*p*-value is based on Chi-square or fisher exact test wherever the expected cell counts are < 5

### EPDS and antenatal depression prevalence

The median (IQR) score for depression symptoms was 4(0–5), with the tool demonstrating high reliability (α = 0.74) for measuring participant depression status. Sixty-three participants had an EPDS score between 12 and 16, giving a prevalence of possible depression of 6.9% (95%CI: 5.3, 8.7%). Of these, 16 (4.7%) and 47(8.1%) were in their second and third trimesters respectively. In the multivariable linear mixed effects regression model, the following characteristics independently predicted a higher odds of depression: rarely discussed and agreed on things with their partner (*p* < 0.001), difficulty accessing food for their family in the last 3 months (*p* = 0.001), unplanned pregnancy (*p* < 0.001), a fear of giving birth for the current pregnancy (p = 0.001), a history of common mental health disorders before being pregnant (*p* < 0.001), and poor social and partner support (both, *p* < 0.001). These factors were therefore included in the path analysis of depression.

### Measurement model for depression and social support

A confirmatory factor analysis showed that a measurement model gives a good fit (RMSEA = 0.042, CFI = 0.99, TLI = 0.98, SRMR = 0.016, coefficient of determination (R^2^ = 0.81). All the factor loadings were found significant at *p* < 0.001. A standardized factor loading for the structural model is displayed in Fig. 2.

### Structural equation model (SEM) for elucidating stress-process model for antenatal depression

The estimated standardized path loadings for the structural equation model are shown in Fig. 2. The overall model fit was excellent according to the various fit indices (RMSEA = 0.037, CFI = 0.98, TLI = 0.97, SRMR = 0.022, coefficient of determination (R^2^ = 0.35)). In line with the stress process model, several direct stressors and mediators related to participants’ risk of antenatal depression were identified (Table 4). Worrying about food for the last 3 months, a history of a common mental health disorder, a fear of giving birth for the current pregnancy, and an unplanned pregnancy were all identified as stressors directly associated with participants’ antenatal depression score (all *p* < 0.001). In addition, social support, marital status, and partner support appeared to partially mediate the effect of identified stressors on antenatal depression. Together, these factors explained 35% of the total variation in depression symptoms (R^2^ = 0.35).

**Table 4:** Direct, Indirect and total effect of stressors and mediators on antenatal depression score among study participants (*N* = 916), Gondar town, Ethiopia, 2018

Pathways	Direct effect β, SE	Indirect effect β, SE	Total effect β, SE
Marital agreement never (1)---rarely(2)---sometimes(3)---most of the time (4)	−0.20(0.055)^a^	−0.08(0.008)^a^	−0.28(0.056)^a^
Partner support Never(1)---rarely(2)---some of the time (3) ---most of the time(4)---always (5)	−0.14(0.041)^a^	−0.04(0.009)^a^	−0.18(0.041)^a^
Social support scale (0---14)	−0.21(0.081)^a^	–	−0.21(0.081)^a^
Adequate food access in the last 3 months			
Yes	−0.11(0.181)^a^	−0.04(0.07)^b^	−0.15(0.191)^a^
Intention for the current pregnancy			
Unplanned	0.15(0.995)^a^	0.11(0.05)^a^	0.26(0.103)^a^
Fear of giving birth to the current pregnancy			
Yes	0.29(0.082)^a^	0.01(0.027)	0.30(0.087)^a^
History of common mental health disorder			
Yes	0.18(0.135)^a^	−0.01(0.032)	0.17(0.137)^a^

^a^ ≤ 0.001, ^b^ < 0.01, β is standardized estimate

### Direct, indirect and total effect of stressors and mediators on antenatal depression

As shown in Fig. 2 and Table 4 (below) marital agreement had both a direct (standardized β = − 0.20) and an indirect (standardized β = − 0.08) negative effect on antenatal depression score. As the level of marital agreement increased by one standard deviation (from low to high), mother’s estimated depression score decreased by 0.28 standard deviations (i.e. total standardized effect β = 0.28). Partner support had both direct (standardized β = − 0.14) and indirect (standardized β = − 0.04) negative effects on antenatal depression score. As partner support increased by one standard deviation (from low to high), the mother’s estimated depression score decreased by 0.18 standard deviations (total standardized effect = − 0.18).

**Fig. 3:**
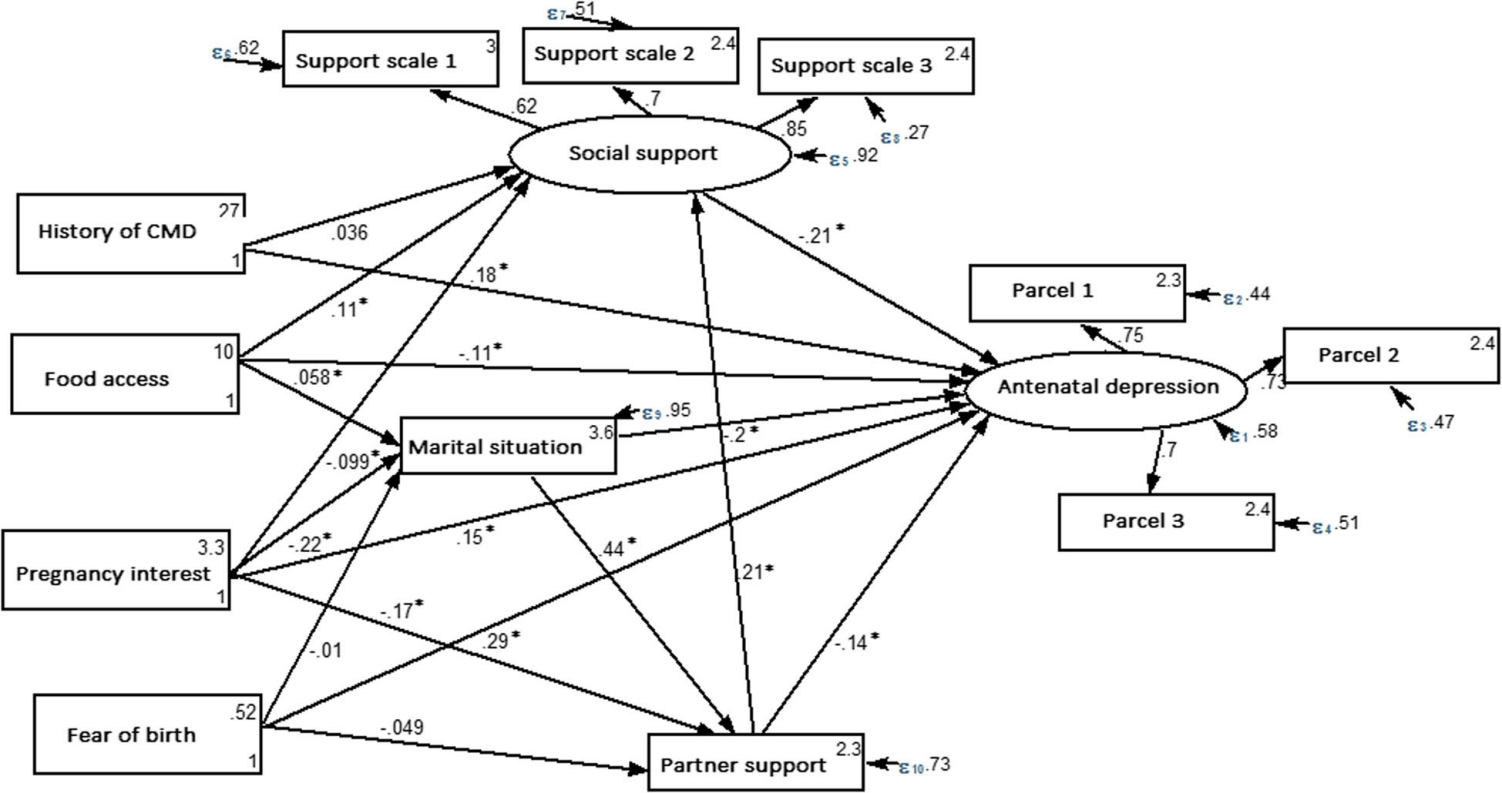
Structural equation modeling for testing a stress process model for antenatal depression (*N* = 916), in Gondar town, Northwest Ethiopia

There was a direct negative (standardized β = − 0.21) relationship between social support and antenatal depression score; so that increasing social support score by one standard deviation would cause a decrease in antenatal depression score by 0.21 standard deviations (total standardized β = − 0.21). In general, increased support from either a partner or other social networks lowered the participants’ depression score. These three variables (marital relationship, social support and partner support) each partially mediated the relationship between stressor variables and antenatal depression. (Table 4 & Fig. 3).

Adequate family food access in the last 3 months had both a direct (standardized β = − 0.11) and an indirect (standardized β = − 0.04) negative effect on antenatal depression score. Adequate food access was inversely associated with antenatal depression and via the joint mediation of social support and marital agreement this association increased (total standardized β = − 0.15). As such, depression score was decreased by 0.15 standard deviation for participants who had adequate food access compared to those who worried about food for their family. Unplanned pregnancy had both a direct (standardized β =0.15) and an indirect (standardized β =0.11) positive effect on antenatal depression score; meaning depression scores in unplanned pregnancy was increased by 0.26 standard deviations (total standardized β = 0.26) compared to other participants.

Participants who feared giving birth for the current pregnancy had a direct positive effect (standardized β = 0.29) on antenatal depression score, with an increased depression score of 0.30 standard deviations (total standardized β = 0.30) above those who had no fear of giving birth. A history of common mental health disorder before pregnancy had a direct positive effect (standardized β = 0.18) on antenatal depression, with an increased depression score of 0.17 standard deviations (total standardized β = 0.17) above those with no history of common mental health disorders. (Table 4 & Fig. 3).

## Discussion

This study utilised a structural equation modelling approach with data from pregnant women in Gondar Town to provide strong support for the conceptual stress process model of the relationships between stressors and antenatal depression developed by Pearlin et al. [45, 80]. In particular, our study showed that stressors and risk factors such as worrying about food for the last 3 months, a history of a mental health disorder, a fear of giving birth for the current pregnancy, and an unplanned pregnancy (through the partial mediation of social support, marital situation, and partner support each) each help determine the likelihood of mothers developing a higher risk of depression during pregnancy.

In this study, 6.9% of participants in their second and third trimester had depression with the highest risk being in the third trimester of pregnancy. This prevalence was slightly lower than that estimated for Debretabor Town, which had a very similar context to the current study (11.8%), [62] and for the rural areas of Jimma (10.8%) [81]. However, the current study excluded participants with an EPDS score above 17 for ethical reasons and only included mothers in their second and third trimesters.

Our estimate for depression prevalence may also have been lower than previous estimates in other regions of Ethiopia [41, 43, 63, 65, 82–84] due to other methodological differences. All other studies included pregnant women irrespective of their week of pregnancy and depression symptoms are believed to be higher in the first trimester than the second and third. Different tools were also used for measuring depression (the Patient Health Questionnaire (PHQ) and the Beck depression inventory (BDI)) with different cut off values defining depression status. Further, most of them were facility-based studies rather than community-based studies, introducing the potential for Berkson’s bias that might lead to over or underestimates of the true prevalence. The current study included the application of an electronic based data collection system, which has been reported to produce better data quality and completeness than paper-based systems as were indicated in previous study [85]. Factors associated with the occurrence of antenatal depression (such as low family income, unplanned pregnancy, poor social support, and a history of pregnancy complications) were also higher in the previous studies [86–89]. Together however, these studies demonstrate that antenatal depression should be considered as a major health threat given the significant negative effects on the mother together with poor developmental trajectories of the fetus [90].

Financial difficulty emerged as an important stressor that had both direct and indirect effects on antenatal depression in the current study. Further, the median depression score was significantly lower for participants with adequate family food access compared to participants with no food access. These findings support path analysis models in China and elsewhere in Ethiopia, where financial strain had both direct and indirect effects on antenatal depression [40, 46]. This finding has important significance in patriarchal societies such as Ethiopia where males typically have the responsibility for generating household income. For example, in this study, more than 70% of the women were occupied with unpaid home duties with complete financial reliance on partners and for which, a failure to provide such a support could pose a significant threat for maternal psychological health [91]. Addressing such societally entrenched issues may represent a question of female empowerment and gender equality and might not be easily or quickly addressed. However, other approaches including expansion of family planning services and institution-based psychotherapy, might be considered useful short-term interventions.

A history of a common mental health disorder was a stressor with a direct and positive association with depression during pregnancy. Due to the familial and recurrent nature of depression and other mental health co-morbidities [92], depression during pregnancy is more likely to exist in women with depression before pregnancy or a family history of mental health morbidity [93]. Recall of prior experiences with depression can also increase the risk of depression [94, 95]. Taken together, this suggests a need for pre-pregnancy screening for depression with a provision of interventions where necessary.

Fear of giving birth had a direct and positive relationship with the occurrence of depression during pregnancy. It has been stated that, women who have a fear of giving birth are more likely to show signs of depression as a result of reduced self-esteem, lower personal coping ability and psychological preparedness for delivery [96]. Indirectly, fear of giving birth might be related to poor obstetric history, lack of experience, unplanned pregnancy, or poor marital relationships – factors which, separately or concomitantly, result in increased maternal stress [97–99]. As with other potential stressors, the sources of fear concerning delivery should be identified early and intervention modalities specific to the cause of the fear established as part of the antenatal care.

In this conceptual model of stress, we also confirmed that having an unplanned pregnancy had both direct and indirect positive effects on antenatal depression. Additionally, poor personal and social resources were predictors of depression. Unwanted or unplanned pregnancy as a risk factor for antenatal depression has been previously demonstrated in Ethiopia [43, 62, 63, 83, 92, 100]. Unplanned pregnancies could directly and negatively affect the three social capitals (namely partner support, relationship quality and social support), significantly reduce their buffering role and increase the risk of developing depression. To mitigate this risk, expanding family planning services to reduce unplanned pregnancies and giving due attention to the mental wellbeing of pregnant mothers with a history of unplanned pregnancy would help alleviate the short- and long-term consequences of antenatal depression.

Similar to previous studies [25, 42, 88, 89, 92, 101], we found that social resources such as good marital conditions, partner and social support mediated a weaker association between stressors and antenatal depression. Good marital relationships also had a synergistic effect since they increased partner support, thereby further enhancing social support and risk of depression. In contrast, a lack of partner or social support has been stated to give rise to a sense of worthlessness and hopelessness, a stage of depression that could ultimately result in maternal suicide or irreversible psychiatric conditions [102]. In summary, a high level of marital agreement, and/or partner and social support significantly reduced the risk of depression, while their absence (in addition to other stressors) increased the risk, consistent with findings from an Ethiopian community-based cohort study in Jimma [81]. Together, these findings support the psychosocial stress theories that acknowledge social and partner support as mediators of a reduced risk of depression and anxiety symptoms [103–105].

### Study limitations and strengths

While there was an ethical imperative to do so, exclusion of study participants with high depression score (≥ 17 on EPDS) would have likely led to an underestimation of the true prevalence of antenatal depression in this community. In addition, since the collected data was cross-sectional, associations may not be causal.

The requirement to reduce participant burden and distress resulted in the omission of variables such as violence and a history of abuse. This, and the relatively low reliability of the stress coping scale, could have resulted in residual confounding and measurement error. Whilst the data and model fit provides empirical support, we cannot rule out a range of possible alternatives to the current stress-process model [106]. Nonetheless, this study is the first of its kind to replicate the stress-process model developed by Pearln et al. [45] and was supported by a large sample size and excellent model fit for this population of pregnant women living in urban areas of Ethiopia.

This study has implications for preventing and controlling antenatal depression in Ethiopia. The identified stressors played an important role in the risk of depression in our participants. Education for girls and women about contraception, counseling on birth preparedness and complication readiness during antenatal care visits, improving employability and/or income generation capabilities, and assessing depression risk before and during pregnancy might help to eliminate the source of many of the identified stressors. Similarly, since marital condition, social and partner support were found to mediate and buffer the relationship between stressors and depression, promoting activities that preserve such social capitals is important to help maintain the psychological and mental wellbeing of mothers.

## Conclusion

Our data provides further evidence for a high risk of antenatal depression and supports previous studies within Ethiopia. Based on our findings, a history of common mental health disorders, an unplanned pregnancy, fear of giving birth and financial difficulties are the main risk factors for antenatal depression. Marital condition, social and partner support were found to buffer or reduce the risk of antenatal depression. As such promoting activities that preserve such social capitals is important to help maintain the psychological and mental wellbeing of the pregnant mothers.

## Supplementary information

**Additional file 1.** English version questionnaire used to collect data for the study.

## Data Availability

The datasets used and/or analysed during the current study available from the corresponding author on reasonable request as this is part of a PhD work.

## Abbreviations

WHO: World Health Organizations
DSM-IV: Diagnostic and statistical manual of mental disorder
AND: Antenatal depression
EPDS: Edinburgh postnatal depression scale
SBRC: Social and behavioral research ethics committee
ODK: Open data collection kit
OSSS: Oslo social support scale
MUAC: Middle-upper arm circumference
PCI: Perinatal coping inventory
TLT: Tucker Lewis Index
CFI: Comparative fit index
RMSEA: Root mean square error of approximation
PHQ: Patient health questionnaire
BDI: Beck depression inventory

## References

[1] Wadhwa PD, Glynn L, Hobel CJ, Garite TJ, Porto M, Chicz-DeMet A (2002). Behavioral perinatology: biobehavioral processes in human fetal development. Regul Pept.

[2] O’Keane V, Marsh MS. Depression during pregnancy. BMJ. 2007;334. 10.1136/bmj.39189.662581.55 PMC1867919 17494021

[3] Sheila M (2008). Marcus. depression-during-pregnancy-rates-risks-and-consequences-motherisk-update-2008.pdf.

[4] Robertson E, Grace S, Wallington T, Stewart DE (2004). Antenatal risk factors for postpartum depression: a synthesis of recent literature. Gen Hosp Psychiatry.

[5] Stewart DE (2011). Depression during pregnancy. N Engl J Med.

[6] Amiel Castro RT, Pinard Anderman C, Glover V, O’Connor TG, Ehlert U, Kammerer M (2017). Associated symptoms of depression: patterns of change during pregnancy. Arch Women’s Ment Health.

[7] Mukherjee S, Trepka M, Pierre-Victor D, Bahelah R, Avent T (2016). Racial/ethnic disparities in antenatal depression in the United States: a systematic review. Matern Child Health J.

[8] Chatillon O, Even C (2010). Antepartum depression: prevalence, diagnosis and treatment. Encephale..

[9] Mitchell-Jones N, Gallos I, Farren J, Tobias A, Bottomley C, Bourne T (2017). Psychological morbidity associated with hyperemesis gravidarum: a systematic review and meta-analysis. BJOG.

[10] Lau Y. Risk factors associated with antenatal depressive symptomatology among Chengdu Chinese women. Merrick, Joav [Ed] (2013) Alternative medicine yearbook, 2011 (pp 107-121) xxx, 572 pp Hauppauge, NY, US: Nova Biomedical Books; US. 2013:107–21.

[11] Li J, Mao J, Du Y, Morris JL, Gong G, Xiong X (2012). Health-related quality of life among pregnant women with and without depression in Hubei, China. Matern Child Health J.

[12] Bavle AD, Chandahalli AS, Phatak AS, Rangaiah N, Kuthandahalli SM, Nagendra PN (2016). Antenatal depression in a tertiary care hospital. Indian J Psychol Med.

[13] Srinivasan N, Murthy S, Singh AK, Upadhyay V, Mohan SK, Joshi A (2015). Assessment of burden of depression during pregnancy among pregnant women residing in rural setting of Chennai. J Clin Diagn Res.

[14] Husain N, Cruickshank K, Husain M, Khan S, Tomenson B, Rahman A (2012). Social stress and depression during pregnancy and in the postnatal period in British Pakistani mothers: a cohort study. J Affect Disord.

[15] Weng SC, Huang JP, Huang YL, Lee TS, Chen YH (2016). Effects of tobacco exposure on perinatal suicidal ideation, depression, and anxiety. BMC Public Health.

[16] Abujilban SK, Abuidhail J, Al-Modallal H, Hamaideh S, Mosemli O (2014). Predictors of antenatal depression among Jordanian pregnant women in their third trimester. Health Care Women Int.

[17] Moshki M, Cheravi K (2016). Relationships among depression during pregnancy, social support and health locus of control among Iranian pregnant women. Int J Soc Psychiatry.

[18] Padmapriya N, Bernard JY, Liang S, Loy SL, Shen Z, Kwek K (2016). Association of physical activity and sedentary behavior with depression and anxiety symptoms during pregnancy in a multiethnic cohort of Asian women. Arch Women’s Ment Health..

[19] Shidhaye P, Shidhaye R, Phalke V (2017). Association of gender disadvantage factors and gender preference with antenatal depression in women: a cross-sectional study from rural Maharashtra. Soc Psychiatry Psychiatr Epidemiol.

[20] Fisher J, Tran T, Duc Tran T, Dwyer T, Nguyen T, Casey GJ (2013). Prevalence and risk factors for symptoms of common mental disorders in early and late pregnancy in Vietnamese women: a prospective population-based study. J Affect Disord.

[21] Manikkam L, Burns JK. Antenatal depression and its risk factors: an urban prevalence study in KwaZulu-Natal. S Afr Med J. 2012;102. 10.7196/samj.6009 23498042

[22] Målqvist M, Clarke K, Matsebula T, Bergman M, Tomlinson M (2016). Screening for antepartum depression through community health outreach in Swaziland. J Community Health.

[23] Rwakarema M, Premji SS, Nyanza EC, Riziki P, Palacios-Derflingher L (2015). Antenatal depression is associated with pregnancy-related anxiety, partner relations, and wealth in women in northern Tanzania: a cross-sectional study. BMC Womens Health.

[24] Thompson O, Ajayi I (2016). Prevalence of antenatal depression and associated risk factors among pregnant women attending antenatal clinics in Abeokuta north local government area, Nigeria. Depression Res Treat.

[25] Zegeye A, Alebel A, Gebrie A, Tesfaye B, Belay YA, Adane F (2018). Prevalence and determinants of antenatal depression among pregnant women in Ethiopia: a systematic review and meta-analysis. BMC Pregnancy Childbirth..

[26] National forum on early childhood program evaluation. Maternal Depression Can Undermine the Development of Young Children. Working Paper No 82009..

[27] Diego MA, Field T, Hernandez-Reif M, Schanberg S, Kuhn C, Gonzalez-Quintero VH (2009). Prenatal depression restricts fetal growth. Early Hum Dev.

[28] Toth SL, Rogosch FA, Manly JT, Cicchetti D (2006). The efficacy of toddler-parent psychotherapy to reorganize attachment in the young offspring of mothers with major depressive disorder: a randomized preventive trial. J Consult Clin Psychol.

[29] Latendresse G, Wong B, Dyer J, Wilson B, Baksh L, Hogue C (2015). Duration of maternal stress and depression. Nurs Res.

[30] Deyessa N, Berhane Y, Emmelin M, Ellsberg MC, Kullgren G, Hogberg U (2010). Joint effect of maternal depression and intimate partner violence on increased risk of child death in rural Ethiopia. Arch Dis Child.

[31] Campbell SB, Brownell CA, Hungerford A, Spieker SI, Mohan R, Blessing JS (2004). The course of maternal depressive symptoms and maternal sensitivity as predictors of attachment security at 36 months. Dev Psychopathol.

[32] Bonari L, Pinto N, Ahn E, Einarson A, Steiner M, Koren G. Perinatal risks of untreated depression during pregnancy. Can J Psychiatr. 2004;49. 10.1177/070674370404901103 15633850

[33] Dayan J, Creveuil C, Herlicoviez M, Herbel C, Baranger E, Savoye C, et al. Role of anxiety and depression in the onset of spontaneous preterm labor. Am J Epidemiol. 2002;155. 10.1093/aje/155.4.293 11836191

[34] Azale T, Fekadu A, Hanlon C (2016). Treatment gap and help-seeking for postpartum depression in a rural African setting. BMC Psychiatry.

[35] Meltzer-Brody S (2011). New insights into perinatal depression: pathogenesis and treatment during pregnancy and postpartum. Dialogues Clin Neurosci.

[36] Gausia K, Fisher C, Ali M, Oosthuizen J (2009). Antenatal depression and suicidal ideation among rural Bangladeshi women: a community-based study. Arch Womens Ment Health.

[37] Mattes E, McCarthy S, Gong G, van Eekelen JA, Dunstan J, Foster J (2009). Maternal mood scores in mid-pregnancy are related to aspects of neonatal immune function. Brain Behav Immun.

[38] Pawlby S, Hay DF, Sharp D, Waters CS, O'Keane V (2009). Antenatal depression predicts depression in adolescent offspring: prospective longitudinal community-based study. J Affect Disord.

[39] Dunkel SC (2011). Psychological science on pregnancy: stress processes, biopsychosocial models, and emerging research issues. Annu Rev Psychol.

[40] Ayano G, Tesfaw G, Shumet S (2019). Prevalence and determinants of antenatal depression in Ethiopia: a systematic review and meta-analysis. PLoS One.

[41] Ayele TA, Azale T, Alemu K, Abdissa Z, Mulat H, Fekadu A (2016). Prevalence and associated factors of antenatal depression among women attending antenatal Care Service at Gondar University Hospital, Northwest Ethiopia. Plos One.

[42] Belay YA, Moges NA, Hiksa FF, Arado KK, Liben ML (2018). Prevalence of antenatal depression and associated factors among pregnant women attending antenatal Care at Dubti Hospital: a case of pastoralist region in Northeast Ethiopia. Depress Res Treat.

[43] Biratu A, Haile D (2015). Prevalence of antenatal depression and associated factors among pregnant women in Addis Ababa, Ethiopia: a cross-sectional study. Reprod Health.

[44] Howard LM, Molyneaux E, Dennis CL, Rochat T, Stein A, Milgrom J (2014). Non-psychotic mental disorders in the perinatal period. Lancet (London).

[45] Pearlin LI, Aneshensel CS, Phelan JC (1999). The stress process revisited. Handbook of the sociology of mental health.

[46] Li Y, Zeng Y, Zhu W, Cui Y, Li J (2016). Path model of antenatal stress and depressive symptoms among Chinese primipara in late pregnancy. BMC Pregnancy Childbirth.

[47] Liu S, Yan Y, Gao X, Xiang S, Sha T, Zeng G (2017). Risk factors for postpartum depression among Chinese women: path model analysis. BMC Pregnancy Childbirth..

[48] CSA (2007). Population and Housing Census Report: Ethiopia.

[49] Ministry of urban development and construction (2016). Background of gondar town administration.

[50] Gondar town health office. Zonal health office health service plan of 2010 EC (2016/2017GC). Unpublished document. 2016..

[51] Australian government health and research cauncle (2015). National Statement on Ethical Conduct in Human Research 2007 (Updated May 2015).

[52] Cox JL, Holden JM, Sagovsky R (1987). Detection of postnatal depression: development of the 10-item Edinburgh postnatal depression scale. Br J Psychiatry.

[53] University of Washington (2008). Open Data Kit (ODK).

[54] Benchmarking of Mobile Data Collection Solutions - What aspects to consider when choosing a tool/platform Terre des Hommes, CartONG, UNHCR. 2017. http://blog.cartong.org/wordpress/wp-content/uploads/2017/08/Benchmarking_MDC_2017_CartONG_2.pd. Accessed June 2019.

[55] Hanlon C, Medhin G, Alem A, Araya M, Abdulahi A, Hughes M (2008). Detecting perinatal common mental disorders in Ethiopia: validation of the self-reporting questionnaire and Edinburgh postnatal depression scale. J Affect Disord.

[56] Töreki A, Andó B, Keresztúri A, Sikovanyecz J, Dudas RB, Janka Z (2013). The Edinburgh postnatal depression scale: translation and antepartum validation for a Hungarian sample. Midwifery..

[57] Su KP, Chiu TH, Huang CL, Ho M, Lee CC, Wu PL (2007). Different cutoff points for different trimesters? The use of Edinburgh postnatal depression scale and Beck depression inventory to screen for depression in pregnant Taiwanese women. Gen Hosp Psychiatry.

[58] Stewart RC, Umar E, Tomenson B, Creed F (2013). Validation of screening tools for antenatal depression in Malawi-a comparison of the Edinburgh postnatal depression scale and self reporting questionnaire. J Affect Disord.

[59] Pereira AT, Bos SC, Marques M, Maia BR, Soares MJ, Valente J (2011). The postpartum depression screening scale: is it valid to screen for antenatal depression?. Arch Women’s Ment Health..

[60] Chorwe-Sungani G, Chipps J. A systematic review of screening instruments for depression for use in antenatal services in low resource settings. BMC Psychiatry. 2017;17(1). 10.1186/s12888-017-1273-7 PMC5366121 28340609

[61] Tesfaye M, Hanlon C, Wondimagegn D, Alem A. Detecting postnatal common mental disorders in Addis Ababa, Ethiopia: validation of the Edinburgh postnatal depression scale and Kessler scales. J Affect Disord 2010;122(1–2):102–8. 10.1016/j.jad.2009.06.020 19615753

[62] Bisetegn TA, Mihretie G, Muche T. Prevalence and predictors of depression among pregnant women in debretabor town, northwest Ethiopia. PLoS One. 2016;11(9). 10.1371/journal.pone.0161108 PMC5019395 27618181

[63] Dibaba Y, Fantahun M, Hindin MJ. The association of unwanted pregnancy and social support with depressive symptoms in pregnancy: evidence from rural southwestern Ethiopia. BMC Pregnancy Childbirth. 2013;13. 10.1186/1471-2393-13-135 PMC3716614 23800160

[64] Meltzer H, Nosikov A, Gudex C (2003). Evelopment of a common instrument for mental health. EUROHIS: developing common instruments for health surveys.

[65] Bitew T, Hanlon C, Kebede E, Medhin G, Fekadu A (2016). Antenatal depressive symptoms and maternal health care utilisation: a population-based study of pregnant women in Ethiopia. BMC Pregnancy Childbirth.

[66] M-t V, Antierens A, Sackl A, Staderini N, Captier V. Which Anthropometric Indicators Identify a Pregnant Woman as Acutely Malnourished and Predict Adverse Birth Outcomes in the Humanitarian Context? PLoS Curr. 2013;5 ecurrents.dis.54a8b618c1bc031ea140e3f2934599c8. 10.1371/currents.dis.54a8b618c1bc031ea140e3f2934599c8 PMC3682760 23787989

[67] WHO (2010). Global recommendations on physical activity for health.

[68] Healthline (2017). How Much Caffeine in a Cup of Coffee? A Detailed Guide.

[69] NHS (2018). Should I limit caffeine during pregnancy?.

[70] Yali AM, Lobel M (1999). Coping and distress in pregnancy: an investigation of medically high risk women. J Psychosom Obstet Gynaecol.

[71] Yali AMLM (2002). Stress-resistance resources and coping in pregnancy. Anxiety Stress Coping.

[72] Dean AG, Sullivan KM, Soe MM (2010). Epi info and OpenEpi in epidemiology and clinical medicine: health applications of free software: CreateSpace.

[73] Hox JJ, Bechger TM (1998). An introduction to structural equation modeling.

[74] Hox JJ, Moerbeek M, Van de Schoot R. Multilevel analysis: techniques and applications: Routledge; 2017.

[75] Little TD, Cunningham WA, Shahar G, Widaman KF (2002). To parcel or not to parcel: exploring the question, weighing the merits. Struct Equ Model.

[76] Matsunaga M (2008). Item parceling in structural equation modeling: a primer. Commun Methods Meas.

[77] Satorra A, Bentler PM (2001). A scaled difference chi-square test statistic for moment structure analysis. Psychometrika..

[78] Norman GR. Path analysis and structural equation modeling. Biostatistics. 2008:211–28.

[79] Kline RB. Principles and practice of structural equation modeling. 2nd ed: Guilford Publications; 2005.

[80] Pearlin LI, Menaghan EG, Lieberman MA, Mullan JT (1981). The stress process. J Health Soc Behav.

[81] Woldetensay YK, Belachew T, Biesalski HK, Ghosh S, Lacruz ME, Scherbaum V (2018). The role of nutrition, intimate partner violence and social support in prenatal depressive symptoms in rural Ethiopia: community based birth cohort study. BMC Pregnancy Childbirth..

[82] Assefa GW (2015). Prevalence and factors associated with antenatal depression among women following antenatal care at Shashemane health facilities, South Ethiopia. Ann Global Health.

[83] Mossie TB, Sibhatu AK, Dargie A, Ayele AD (2017). Prevalence of antenatal depressive symptoms and associated factors among pregnant women in Maichew, North Ethiopia: an institution based study. Ethiop J Health Sci.

[84] Sahile MA, Segni MT, Awoke T, Bekele D (2017). Prevalence and predictors of antenatal depressive symptoms among women attending Adama hospital antenatal clinic, Adama, Ethiopia. Int J Nurs Midwifery.

[85] Macharia P, Muluve E, Lizcano J, Cleland C, Cherutich P, Kurth A, editors. Open data kit, a solution implementing a mobile health information system to enhance data management in public health. 2013 IST-Africa Conference & Exhibition; 2013 29–31 May 2013.

[86] Chou F-H, Kuo S-H, Wang R-H. A Longitudinal Study of Nausea and Vomiting, Fatigue and Perceived Stress in, and Social Support for, Pregnant Women Through the Three Trimesters. Kaohsiung J Med Sci. 24(6):306–14. 10.1016/S1607-551X(08)70157-8 PMC11917605 18635416

[87] Fisher J, Cabral de Mello M, Patel V, Rahman A, Tran T, Holton S (2012). Prevalence and determinants of common perinatal mental disorders in women in low- and lower-middle-income countries: a systematic review. Bull World Health Organ.

[88] Sparling TM, Henschke N, Nesbitt RC, Gabrysch S. The role of diet and nutritional supplementation in perinatal depression: a systematic review. Mat Child Nutr. 2017;13(1). 10.1111/mcn.12235 PMC6865932 26840379

[89] Stuart-Parrigon K, Stuart S (2014). Perinatal depression: an update and overview. Curr Psychiatry Rep.

[90] O’Hara MW, Segre LS. Psychological disorders of pregnancy and the post partum. In R.S. Gibbs, B.Y. Karlan, A.F. Haney, & I. Nygaard (Eds.), Danforth’s obstetrics and gynecology 10. Philadelphia: Lippincott, Williams & Wilkins. 2008.

[91] Golberstein E (2015). The effects of income on mental health: evidence from the social security notch. J Ment Health Policy Econ.

[92] Getinet W, Amare T, Boru B, Shumet S, Worku W, Azale T (2018). Prevalence and risk factors for antenatal depression in Ethiopia: systematic review. Depress Res Treat.

[93] Shyn SI, Hamilton SP (2010). The genetics of major depression: moving beyond the monoamine hypothesis. Psychiatric Clin North Am.

[94] Montgomery E, Pope C, Rogers J (2015). The re-enactment of childhood sexual abuse in maternity care: a qualitative study. BMC Pregnancy Childbirth.

[95] Leeners B, Stiller R, Block E, Görres G, Rath W, Tschudin S. Prenatal care in adult women exposed to childhood sexual abuse. J Perinat Med. 2013:365. 10.1515/jpm-2011-0086 23314504

[96] Izadirad H, Niknami S, Zareban I, Hidarnia A (2017). Effects of social support and self-efficacy on maternal prenatal cares among the first-time pregnant women, Iranshahr, Iran. J Family Reprod Health.

[97] Roomruangwong C, Epperson CN (2011). Perinatal depression in Asian women: prevalence, associated factors, and cultural aspects. Asian Biomed.

[98] Underwood L, Waldie K, D’Souza S, Peterson ER, Morton S (2016). A review of longitudinal studies on antenatal and postnatal depression. Arch Women’s Ment Health..

[99] Underwood L, Waldie KE, D’Souza S, Peterson ER, Morton SMB (2017). A longitudinal study of pre-pregnancy and pregnancy risk factors associated with antenatal and postnatal symptoms of depression: evidence from growing up in New Zealand. Matern Child Health J.

[100] Duko B, Ayano G, Bedaso A (2019). Depression among pregnant women and associated factors in Hawassa city, Ethiopia: an institution-based cross-sectional study. Reprod Health.

[101] Gelaye B, Rondon MB, Araya R, Williams MA (2016). Epidemiology of maternal depression, risk factors, and child outcomes in low-income and middle-income countries. Lancet Psychiatry.

[102] Liabsuetrakul T, Vittayanont A, Pitanupong J (2007). Clinical applications of anxiety, social support, stressors, and self-esteem measured during pregnancy and postpartum for screening postpartum depression in Thai women. J Obstet Gynaecol Res.

[103] Ford E, Ayers S (2009). Stressful events and support during birth: the effect on anxiety, mood and perceived control. J Anxiety Disord.

[104] Jeong H-G, Lim J-S, Lee M-S, Kim S-H, Jung I-K, Joe S-H (2013). The association of psychosocial factors and obstetric history with depression in pregnant women: focus on the role of emotional support. Gen Hosp Psychiatry.

[105] Aktan NM (2012). Social support and anxiety in pregnant and postpartum women: a secondary analysis. Clin Nurs Res.

[106] Little TD, Card NA, Bovaird JA, Preacher KJ, Crandall CS (2007). Structural equation modeling of mediation and moderation with contextual factors. Model Contextual Effect longitudinal Stud.

